# Supplementary material for: Mapping the neutralizing specificity of human anti-HIV serum by deep mutational scanning
Source: Cell Host Microbe. 2023 Jul 12;31(7):1200–1215.e9. doi: 10.1016/j.chom.2023.05.025 (PMC10351223; doi:10.1016/j.chom.2023.05.025)
Supplement: Document S1. Figures S1–S3 and Table S1 [file mmc1.pdf]

Cell Host & Microbe, Volume *31*

Supplemental information

Mapping the neutralizing specificity of human anti-HIV serum by deep mutational scanning

Caelan E. Radford, Philipp Schommers, Lutz Gieselmann, Katharine H.D. Crawford, Bernadeta Dadonaite, Timothy C. Yu, Adam S. Dingens, Julie Overbaugh, Florian Klein, and Jesse D. Bloom

Supplemental Figure 1:

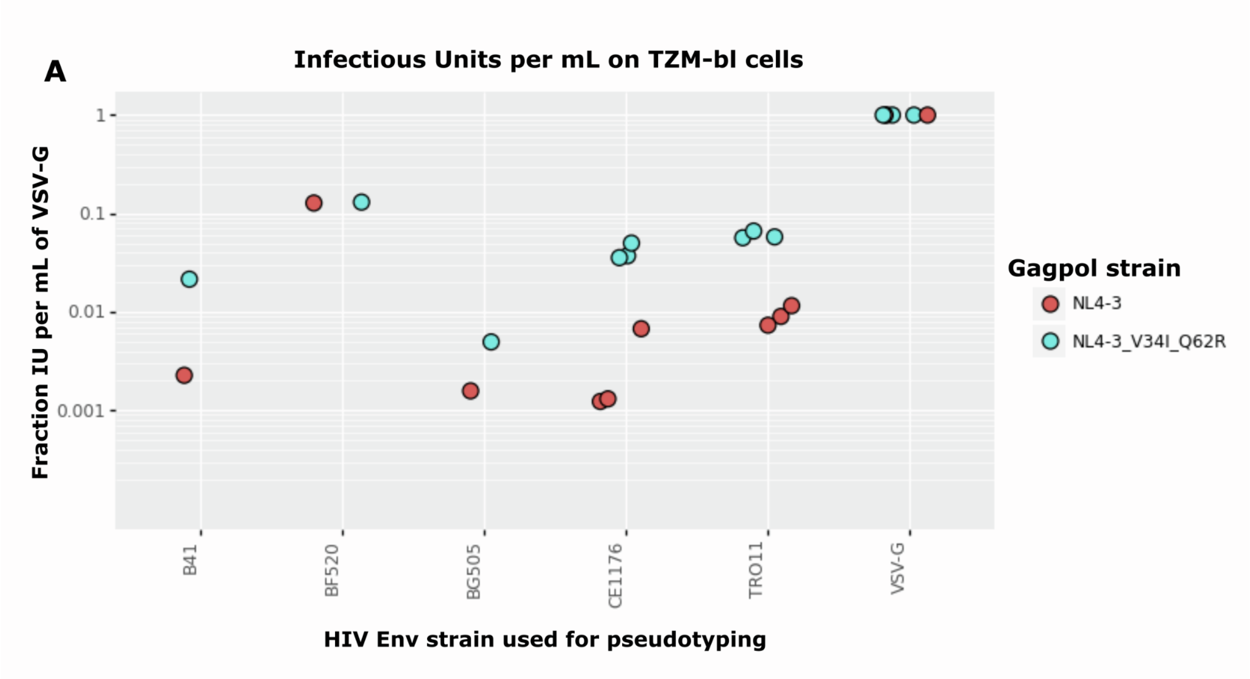

Supplemental Figure 1: Titers of Env or VSV-G pseudotyped lentiviruses on TZM-bl cells, related to Figure 1.

Lentiviruses were produced by transfecting 293T cells with the same ZsGreen reporter lentiviral backbone, Rev, and Tat expressing plasmids for each condition, along with an HIV Env from the indicated viral strain or a VSV-G expressing plasmid, and either a NL4-3 or NL4-3 V34I Q26R Gag-Pol expressing plasmid as indicated. Data are from different virus preparations and titering dates. The infectious units per mL on TZM-bl cells were normalized to VSV-G infectious units per mL by dividing each condition's infectious units per mL by that of the VSV-G pseudotyped virus with the same Gag-Pol that was produced and titered on the same dates, performing this normalization to correct for batch effects. The titers for the VSV-G pseudotyped viruses ranged from ~1.5-35 million infectious units per mL. Gag-Pol mutations V34I and Q62R were made based on previous studies that showed these mutations can rescue Env incorporation deficiencies[66,67].

Supplemental Figure 2:

A

	398F1	246F3	CNE55	CNE8	X2278	Tro11	BJOX2000	CH119	CE1176	CE0217	25710	X1632	BF520	MLV	Mean Neutr.	Breadth (%)
	A	AC	AE	AE	B	B	BC	BC	C	C	C	G	A	NA		
**IDC#0508**	42	50	41	36	85	71	399	71	229	127	94	120	28	>500	86	100
**IDC#0513**	136	281	140	102	75	85	277	174	271	>500	96	233	337	>500	152	92
**IDC#0561**	171	85	124	60	16	38	42	114	453	111	69	94	49	>500	83	100
**IDF#033**	25	36	69	72	52	28	179	207	62	63	66	188	60	>500	69	100

B

Patient ID	Country	Gender	Ethnicity	CDC Status	Viral load at blood draw	CD4 Count	On ART?	Virus Clade	Years since HIV-1 Diagnosis	Age (years)
IDC#0508	Germany	M	Middle Europe	A2	118000	450	No	D	8.91	37
IDC#0513	Germany	M	Middle Europe	NA	42	445	Yes	B	30.66	66
IDC#0561	Germany	M	Middle Europe	A2	5530	550	No	B	20.19	48
IDF#033	Germany	F	Central/South Africa	B3	19	313	Yes	D	20.38	53

C

	VRC01-like	b12-like	HJ16-like	8ANC195-like	PG9-like	PGT128-like	2F5-like	10E8-like	35O22-like	PGT151-like	Neut breadth (%)
**IDC#0508**	0.71	0.10			0.17					0.02	90%
**IDC#0513**	0.40			0.13	0.23			0.24			70%
**IDC#0561**	0.77		0.06				0.04		0.13		75%
**IDF#033**	0.33		0.14	0.04		0.32	0.11		0.05		60%

Supplemental Figure 2: Broadly neutralizing human anti-HIV sera, related to Figures 4, 5, 6, and 7.

(A) Neutralization of a global HIV panel by each serum[45]. Values reported are IC50 in ug/mL for purified IgGs. (B) Clinical data related to the individual from whom each serum was collected. (C) f61 neutralization fingerprinting results for each sera[14].

Supplemental Figure 3:

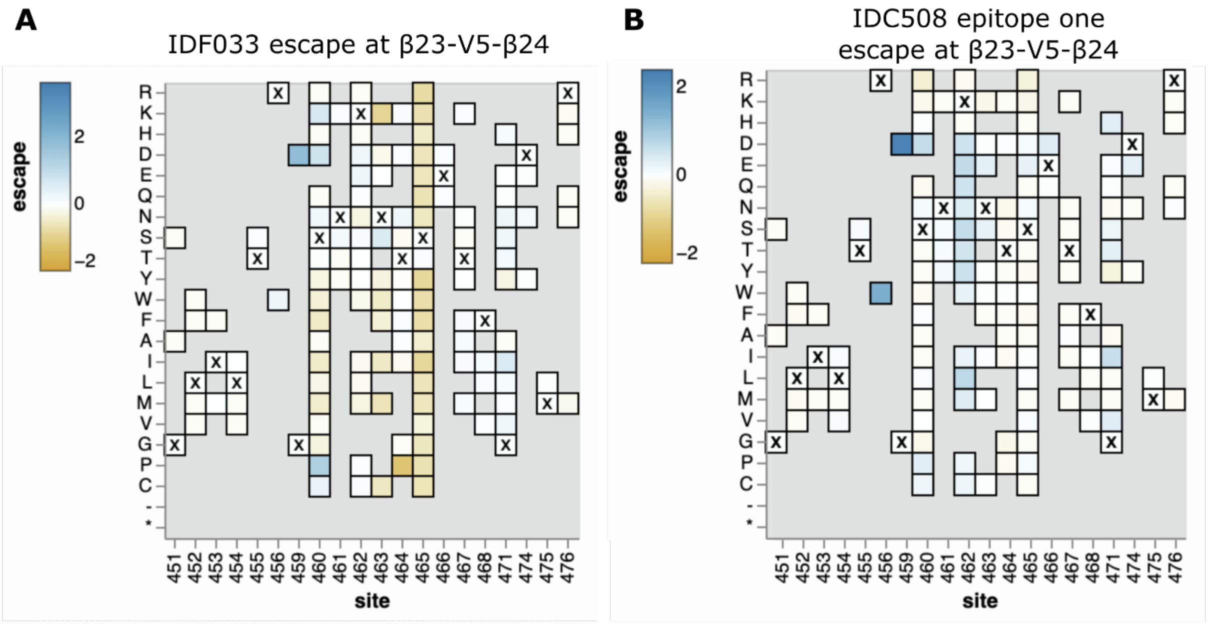

Supplemental Figure 3: Zoomed in views of mutation-level escape at some key sites for IDF033 and IDC508, related to Figure 6.

(A) Heatmap of escape of individual mutations in β23-V5-β24 for IDF033. Residues marked with X are wildtype residues in BF520. Residues grayed out are not present in the variant libraries. (B) Heatmap for IDC508 epitope 1. See https://dms-vep.github.io/HIV_Envelope_BF520_DMS_CD4bs_sera/IDF033_escape_plot.html and https://dms-vep.github.io/HIV_Envelope_BF520_DMS_CD4bs_sera/IDC508_escape_plot.html for interactive versions of the full Env escape maps for IDF033 and IDC508, respectively.

Supplemental Table 1:

Supplemental table 1: Primers and sequences related to Figure 1.

Primers for linearizing BF520 sequences	
Forward linearizing primer (VEP_amp_for_long)	AcgcgCAGCCGAGCCACATCGCTC
Reverse linearizing primer (lin_rev_BF520)	GGAAAGAGTGTgatccaactaggcgc
Primers for mutagenizing BF520 sequences	
Forward mutagenic primer pool	https://github.com/dms-vep/HIV_Envelope_BF520_DMS_CD4bs_sera/blob/main/library_design/results/primers/IDT_BF520_primers_for.csv
Reverse mutagenic primer pool	https://github.com/dms-vep/HIV_Envelope_BF520_DMS_CD4bs_sera/blob/main/library_design/results/primers/IDT_BF520_primers_rev.csv
Primers for barcoding BF520 sequences	
Forward linearizing primer (VEP_amp_for_long)	AcgcgCAGCCGAGCCACATCGCTC
Reverse barcoding primer (BC_BF520_long)	tatgtaacgcggaactccactaggaacatttctctctcgaaTCTAGANNNNNNNNNNNNNNNNAGATCGGAAGAGCGTCGTGTAGGGAAAGAGTGTgatccaactaggcgc
PacBio nucleotide tagging primer set one	
5′ nucleotide tagging primer (PacBio_5pri_G)	ctagccattcagagGCAGCCGAGCCACgTCGCTC
3′ nucleotide tagging primer (PacBio_3pri_C)	CGCTCAACCAGTACGAGCCGTAAGTTATGTAACGCGGAACTCCACcAGGAAC
PacBio nucleotide tagging primer set two	
5′ nucleotide tagging primer (PacBio_5pri_C)	ctagccattcagagGCAGCCGAGCCACcTCGCTC

3′ nucleotide tagging primer (PacBio_3pri_G)	CGCTCAACCAGTACGAGCCGTAAGTTATGTAACGCGGAACTCCACgAGGAAC
PacBio round two primers	
5′ PacBio round 2 forward primer (PacBio_5pri_RND2)	CTAGCCATTCAGAGGCAGCCGAG
3′ PacBio round 2 forward primer (PacBio_3pri_RND2)	CGCTCAACCAGTACGAGCCGTAAGTTATGTAAC
Round one Illumina barcode sequencing preparation primers	
Illumina round 1 forward primer (IlluminaRnd1_For)	CTCTTTCCCTACACGACGCTCTTCCGATCT
Illumina round 1 reverse primer (IlluminaRnd1_rev3)	CTGGAGTTCAGACGTGTGCTCTTCCGATCTgtccctattggcgttactatgggaacatacgtc
Round two Illumina barcode sequencing preparation primers	
Illumina round 2 universal forward primer (Rnd2ForUniversal)	AATGATACGGCGACCACCGAGATCTACACTCTTTCCCTACACGACGCTCTTCCGATCT
Illumina round 2 indexing reverse primer (Indexing primers)	CAAGCAGAAGACGGCATACGAGATxxxxxxxxGTGACTGGAGTTCAGACGTGTGCTCTTCCGATCT<br><br>Where “xxxxxxxx” are NextFlex indices unique to each primer
